# Systemic immune-inflammation index and its relation to blood pressure and dyslipidemia in adults: A retrospective study

**DOI:** 10.1097/MD.0000000000038810

**Published:** 2024-07-12

**Authors:** Ghadeer S. Aljuraiban, Fahad J. Alharbi, Ali O. Aljohi, Abdullah Z. Almeshari, Abdulaziz S. Alsahli, Bader Saad Alotaibi, Manal Abudawood, Waad Alfawaz, Mahmoud Abulmeaty

**Affiliations:** a Department of Community Health Sciences, College of Applied Medical Sciences, King Saud University, Riyadh, Saudi Arabia; b Department of Central Military Laboratory & Blood Bank, Prince Sultan Military Medical City, Riyadh, Saudi Arabia; c Department of Clinical Laboratory Sciences, College of Applied Medical Sciences, King Saud University, Riyadh, Saudi Arabia; d Deputyship of Research Chairs, Deanship of Scientific Research, King Saud University, Riyadh, Saudi Arabia; e Central Research Laboratory, King Saud University, Riyadh, Saudi Arabia.

**Keywords:** blood pressure, hypertension, inflammation, systemic immune-inflammation index

## Abstract

High blood pressure (BP) and dyslipidemia are major risk factors for cardiovascular disease mortality. The systemic immune-inflammation index (SII) has been suggested as a predictive tool to identify those at risk for chronic diseases, however, its use for predicting high BP and dyslipidemia has not been thoroughly investigated. This study aimed to examine the association between SII and high BP as well as lipid markers. Retrospective hospital data from a large cohort (n = 3895) of Saudi adults aged ≥18 years were analyzed. Lipid markers (cholesterol, high-density lipoprotein, low-density lipoprotein [LDL]), systolic BP, and diastolic BP measures were extracted. When the sample was divided into quartiles of SII, cholesterol, triglycerides, and LDL were higher in those with a higher SII than in those with a lower SII (*P* < .01). After adjusting for potential confounders, higher SII was significantly associated with higher odds of hypertension (odds ratio: 1.12, 95% confidence interval: 1.04–1.21) and elevated LDL (odds ratio: 1.07, 95% CI: 1.02–1.14), but not with elevated cholesterol. Across quartiles of SII, there was a significant trend between higher SII and the odds of hypertension in people with diabetes and those aged ≥65 years. The SII could be an economical predictive measure for identifying individuals at risk of hypertension and some aspects of dyslipidemia. Longitudinal studies are needed to confirm this relationship.

## 1. Introduction

Cardiovascular disease is the leading cause of mortality from noncommunicable diseases worldwide, accounting for 17.8 million deaths.^[1]^ The global burden of cardiovascular disease is staggering, and this burden extends to Saudi Arabia, where over 200,000 people lived with cardiovascular disease as of 2016.^[2]^ Dyslipidemia and high blood pressure are critical cardiovascular risk factors.^[3,4]^ While the mean blood pressure has not necessarily increased in the past 40 years, the rates of hypertension continue to rise, especially in some areas of the world.^[5]^ In the past 30 years, the rates of dyslipidemia have increased.^[6]^ Despite these high rates, it has been estimated that nearly half (45.6%) of people are unaware of their hypertension; even fewer receive treatment (36.9%), and only a fraction of these achieve blood pressure control (13.8%).^[7]^ Similarly, individuals impacted with dyslipidemia may also be unaware, especially considering dyslipidemia can be asymptomatic. Thus, the identification of individuals at risk for hypertension and dyslipidemia through affordable and available means is merited.

The systemic immune-inflammation index (SII) is a prognostic and predictive score calculated using platelets, lymphocytes, and neutrophil levels.^[8]^ Initially developed for determining prognoses for patients following surgery for hepatocellular carcinoma,^[8]^ it has since been examined as an inflammatory marker for several health outcomes^[9–23]^ and mortality.^[24–27]^ A recent systematic review and meta-analysis indicated that SII was significantly associated with cardiovascular disease risk (e.g., stroke, myocardial infarction, peripheral arterial disease), and levels of SII were significantly higher at disease onset than among individuals in the general population.^[12]^ Furthermore, SII is touted as a cost-effective tool, considering its components are often collected as part of routine bloodwork.^[8]^

SII has been examined for its association with hypertension or blood pressure in several cross-sectional studies using data from the United States National Health and Nutrition Examination Survey (NHANES).^[28–30]^ Each study found a positive, but not necessarily linear, relationship between the two.^[28–30]^ Compared to other inflammatory markers (e.g., platelet-to-lymphocyte ratio, lymphocyte-to-monocyte ratio, and neutrophil-to-lymphocyte ratio), Xu et al^[30]^ identify SII as a superior marker that could potentially be used as a warning for early identification of personal inflammatory response. A retrospective study of 91 patients found SII was an independent predictor of nondipper hypertension.^[31]^ Similar studies explicitly assessing the utility of the SII for predicting dyslipidemia are also limited. A cross-sectional study also pulling from NHANES data found a significant positive correlation between SII and dyslipidemia,^[15]^ and a study that incorporated aspects of dyslipidemia in the definition of metabolic syndrome also found a positive relationship.^[16]^ However, additional cohort studies are needed to supplement the previously found cross-sectional results.^[32]^

This study aimed to examine the relationship between SII and cardiovascular risk factors (i.e., high blood pressure and dyslipidemia) in a large retrospective cohort of adults in Saudi Arabia.

## 2. Materials and methods

### 2.1. Study design

This retrospective study gathered data from the Prince Sultan Military Medical City for analysis. A total of 4732 patients were initially recruited. Following the exclusion of 837 individuals due to missing data for specific markers, the total number of participants included was 3895. The Institutional Review Board at Prince Sultan Military Medical City approved the study design and protocols (IRB number: E-2115).

### 2.2. Systemic immune-inflammation index

Fasting blood samples were collected as per protocols laid out by international standards. Regular quality control checks were carried out on all laboratory equipment to ensure the validity and reliability of results. Platelet count and the differential white cell count, including neutrophils, lymphocytes, and monocytes, were analyzed using a hematology analyzer (Siemens Advia 2120) in accordance with the manufacturer’s instruction manual. The following formula was used to compute SII.^[8]^


$$\begin{array}{l} SII=\frac{Platelet\;\;\;count\;\;\;\left(10^{9}/L\right)\times Neutrophil\;\;\;count\;\;\;\left(10^{9}/L\right)}{Lymphocyte\;\;\;count\;\;\;\left(10^{9}/L\right)} \end{array}$$

### 2.3. Other data

Fasting triglycerides, cholesterol, high-density lipoprotein (HDL), low-density lipoprotein (LDL), fasting blood sugar, and insulin levels were measured using the Cobas-8000 autoanalyzer (Roche Diagnostics, Basel, Switzerland). Hemoglobin A1c (HbA1c) level was analyzed using the Cobas-513 autoanalyzer (Roche Diagnostics).

Elevated cholesterol was determined when total blood cholesterol measurement was >200 mg/dL, while elevated LDL-cholesterol was identified when LDL was >190 mg/dL.^[33]^

The patient’s hospital records included diagnoses of type 2 diabetes, prediabetes, and normoglycemia. Type 2 diabetes is diagnosed if the fasting blood glucose level is 126 mg/dL or higher, or if the 2-hour plasma glucose tolerance test result is 200 mg/dL or higher, or if the HbA1c level is 6.5% (48 mmol/mol) or higher, as confirmed by a repeated test on a different day. Prediabetes, a condition where blood glucose level is higher than normal, was indicated by fasting blood glucose levels between 100 and 125 mg/dL, or by 2-hour plasma glucose tolerance test results between 140 and 199 mg/dL, or by HbA1c levels between 5.7% and 6.4% (39–46 mmol/mol). Normoglycemia was defined as a fasting blood glucose level of <100 mg/dL after 8 hours of fasting, or a 2-hour plasma glucose tolerance test result of <140 mg/dL, or an HbA1c level of 5.6% (38 mmol/mol) or less, which indicates the average blood glucose level over the last 2 to 3 months.

### 2.4. Blood pressure measurement

A strict protocol was used to measure blood pressure. It was measured in a calm environment, with patients sitting down in a relaxed position with their feet resting firmly on the ground. Patients were asked to empty their bladders prior to the blood pressure reading. Skilled hospital staff were instructed to take 2 intermittent readings to rule out the chance of false readings. The Omron HEM 705-CP) OMRON Corp., Kyoto, Japan) was used as the standard equipment for all patients. Hypertension was defined as a systolic blood pressure ≥130 mm Hg or a diastolic blood pressure ≥80 mm Hg.^[34]^

### 2.5. Anthropometric data

Weight and height were measured using standard hospital equipment (weighing scale and a portable stadiometer [Marsden H226]). Body mass index (BMI) was calculated by dividing weight (kg) by body height (m^2^). BMI was classified into the following 3 categories: normal (≤25.0 kg/m^2^), overweight (>25.0– ≤ 30.0 kg/m^2^), and obese (>30.0 kg/m^2^).

### 2.6. Statistical analysis

For all statistical analyses, SAS version 9.3 by SAS Institute in Cary, NC was used. Categorical variables were expressed as frequencies and continuous variables were presented as means (standard deviations [SDs]).

We used generalized linear regression (PROC GLM) to calculate age and gender-adjusted means of baseline characteristics stratified by quartiles of SII (*Q*1 < 267.4, *Q*2 ≥ 267.4 and < 400.7, *Q*3 ≥ 400.7 and < 588.5, *Q*4 ≥ 588.5).

Logistic regression analysis was used to estimate the odds of hypertension, elevated LDL, and elevated cholesterol by quartiles and per 1 SD (217.6) increase in SII while adjusting for other variables that could potentially influence this association. Model 1 was adjusted for age (years) and gender (male/female). Model 2 was additionally adjusted for white blood cell count, platelet count, and BMI. Interaction terms were used to examine potential effect modification by gender, BMI, and age.

Additionally, we included subgroup analysis for each outcome of interest where we stratified participants by diabetes status (diabetic/prediabetic/nondiabetic), gender, BMI groups, and age (divided according to the Saudi General Authority of Statistics^[35]^ into ≤24.0 years, 24.1–54.0 years, 54.1–64.0 years, ≥64.1 years). A *P* value < .05 was deemed statistically significant.

## 3. Results

### 3.1. Baseline data

Data analyzed for the current investigation included 3895 participants. Men comprised 40.7% (n = 1585) of total participating adults, while 2310 were women. Approximately 76.0% had normal weight, 10.0% had overweight, and 14% had obesity (Supplementary Table 1, Supplemental Digital Content, http://links.lww.com/MD/N119).

When the sample was divided into quartiles of SII, there was a significant difference in cholesterol, triglycerides, and LDL levels across quartiles (*P* < .01) (Table 1).

**Table 1:** Characteristics stratified by quartiles of systemic immune-inflammation index, n = 3895.*

	SII				*P* value
	*Q*1 < 267.4	*Q*2 ≥ 267.4 and <400.7	*Q*3 ≥ 400.7 and < 588.5	*Q*4 ≥ 588.5	
	Mean (95% CI)	Mean (95% CI)	Mean (95% CI)	Mean (95% CI)	
n	973	974	974	974	
SII (median)	200.9	332.3	477.4	785.0	
Age (yr)	49.6 (48.8–50.5)	48.3 (47.2–49.5)	47.4 (46.2–48.6)	48.1 (46.9–49.3)	.01
BMI (kg/m^2^)	29.6 (28.2–31.1)	31.3 (29.1–33.4)	29.2 (27.1–31.2)	28.4 (26.5–30.3)	.28
SBP (mm Hg)	126.9 (125.8–128.1)	125.7 (124.2–127.2)	127.1 (125.6–128.6)	125.3 (123.9–126.7)	.17
DBP (mm Hg)	74.9 (74.2–75.6)	74.3 (73.4–75.3)	74.1 (73.2–75.1)	73.8 (72.9–74.7)	.31
Cholesterol (mmol/L)	4.6 (4.4–4.5)	4.6 (4.5–4.6)	4.6 (4.5–4.7)	4.6 (4.6–4.7)	.01
Triglycerides (mmol/L)	17.5 (16.7–18.3)	19.5 (18.5–20.6)	19.1 (18.1–20.2)	18.1 (17.0–19.2)	.01
LDL (mmol/L)	2.6 (2.5–2.6)	2.7 (2.6–2.7)	2.7 (2.6–2.7)	2.7 (2.5–2.7)	.001
HDL (mmol/L)	1.3 (1.2–1.3)	1.3 (1.2–1.3)	1.3 (1.2–1.3)	1.2 (1.2–1.3)	.64

BMI = body mass index, CI = confidence interval, DBP = diastolic blood pressure, HDL = high-density lipoprotein, LDL = low-density lipoprotein, SBP = systolic blood pressure, SII = systemic immune-inflammation index.

* Data are presented as mean (95% CI) adjusted for age and gender.

### 3.2. Relationship between SII, hypertension, and lipid markers

Logistic regression analysis showed a significant association between the odds of hypertension and SII (odds ratio [OR]: 1.12, 95% CI: 1.04–1.21) (model 2; Table 2). This relationship prevailed for some subgroups when data were analyzed separately (people with diabetes, aged ≥65 years) (Table 2).

**Table 2:** Odds ratio of hypertension, elevated LDL, and elevated cholesterol by quartiles and per 1 unit increase of SII in a sample of Saudi adults (n = 3895).*†

	*Q*1	*Q*2	*Q*3		*Q*4	*P* for trend	SII by 1 SD (217.6)
SII	<267.3	267.3–400.67		400.67–588.45	>588.45		
Hypertension							
Cases	224/973	220/974		208/974	402/974		1054/3895
Model 1	1.00	1.06 (1.02–1.12)		1.01 (0.94–1.15)	1.13 (1.07–1.26)	.04	1.11 (1.02–1.22)
Model 2	1.00	1.04 (0.99–1.11)		1.06 (0.99–1.15)	1.13 (1.02–1.26)	.04	1.12 (1.04–1.21)
Subgroup analysis							
Normoglycemia (n = 1555)	1.00	1.27 (0.83–1.97)		0.94 (0.60–1.45)	1.04 (0.66–1.60)	.60	1.00 (0.99–1.00)
Cases	64/502	73/324		39/361	44/368		220/1555
Prediabetes (n = 1160)	1.00	0.82 (0.62–1.09)		0.95 (0.71–1.27)	1.01 (0.76–1.35)	.54	1.00 (0.99–1.00)
Cases	198/192	45/324		95/344	104/300		442/1160
Diabetes (n = 1180)	1.00	1.64 (1.16–2.33)		1.51 (1.07–2.13)	1.78 (1.26–2.52)	.002	1.10 (1.04–1.16)
Cases	140/309	102/445		74/220	76/206		392/1180
Normal weight (n = 2825)	1.00	1.21 (0.95–1.56)		1.10 (0.85–1.42)	1.33 (1.03–1.04)	.14	0.95 (0.94–0.96)
Cases	209/546	129/775		111/770	126/734		575/2825
Overweight (n = 426)	1.00	0.84 (0.47–1.50)		1.53 (0.86–2.71)	0.93 (0.54–1.60)	.31	0.95 (0.94–0.97)
Cases	75/110	80/100		66/100	95/116		316/426
Obese (n = 644)	1.00	1.20 (0.76–1.89)		0.94 (0.60–1.47)	0.91 (0.59–1.41)	.74	0.99 (0.98–1.00)
Cases	73/255	93/119		89/127	102/143		357/644
Female (n = 2310)	1.00	1.26 (0.95–1.66)		1.07 (0.81–1.42)	1.34 (1.02–1.76)	.12	0.99 (0.98–1.00)
Cases	177/861	110/418		110/369	124/662		521/2310
Male (n = 1585)	1.00	0.96 (0.73–1.27)		1.14 (0.86–1.51)	1.12 (0.83–1.45)	.65	1.01 (0.99–1.02)
Cases	160/531	225/221		118/332	142/501		645/1585
Age groups (yr)							
≤24	1.00	1.29 (0.49–3.34)		1.30 (0.45–3.39)	2.11 (0.84–5.25)	.46	0.98 (0.98–0.99)
25–54	1.00	0.81 (0.59–1.13)		0.81 (0.58–1.12)	1.02 (0.74–1.41)	.39	0.98 (0.98–0.99)
55–64	1.00	1.20 (0.82–1.75)		1.28 (0.86–1.89)	1.29 (0.85–1.96)	.47	0.99 (0.98–1.00)
≥65	1.00	1.27 (0.86–1.87)		1.49 (1.03–2.14)	1.52 (1.06–2.19)	.05	1.07 (1.01–1.12)
Elevated LDL							
Cases	631/973	371/974		359/974	288/974		1649/3895
Model 1	1.00	0.78 (0.66–0.92)		1.11 (0.92–1.28)	1.15 (1.04–1.35)	.0001	1.08 (1.01–1.13)
Model 2	1.00	0.77 (0.65–0.92)		1.12 (0.92–1.29)	1.13 (1.02–1.36)	.0001	1.07 (1.02–1.14)
Subgroup analysis							
Normoglycemia (n = 1555)	1.00	0.74 (0.55–0.99)		0.98 (073–1.32)	1.09 (0.83–1.45)	.08	1.00 (0.99–1.00)
Cases	182/502	114/324		136/361	104/368		536/1555
Prediabetes (n = 1160)	1.00	1.16 (0.92–1.45)		1.09 (0.85–1.40)	0.72 (0.55–0.93)	.54	1.00 (0.99–1.00)
Cases	289/141	187/245		159/374	123/400		758/1160
Diabetes (n = 1180)	1.00	1.28 (0.91–1.79)		1.00 (0.71–1.42)	1.03 (0.72–1.46)	.54	1.00 (0.99–1.02)
Cases	100/549	106/205		124/220	130/206		460/1180
Normal weight (n = 2825)	1.00	0.76 (0.63–0.93)		1.16 (0.96–1.39)	1.22 (1.01–1.46)	.04	1.05 (1.01–1.16)
Cases	483/546	305/775		292/770	209/734		1289/2825
Overweight (n = 426)	1.00	1.02 (0.58–1.79)		1.07 (0.63–1.82)	1.18 (0.67–2.06)	.05	1.05 (1.00–1.11)
Cases	57/171	27/80		29/78	34/97		147/426
Obese (n = 644)	1.00	1.02 (0.58–1.79)		1.07 (0.63–1.82)	1.18 (0.67–2.06)	.03	1.06 (1.01–1.12)
Cases	91/255	39/119		38/127	45/143		213/644
Female (n = 2310)	1.00	0.84 (0.68–1.04)		1.14 (0.92–1.41)	1.18 (0.95–1.47)	.02	1.08 (1.03–1.15)
Cases	325/486	205/556		218/606	194/662		942/2310
Male (n = 1585)	1.00	0.73 (0.55–0.97)		1.05 (0.82–1.35)	1.11 (0.88–1.43)	.05	1.05 (1.01–1.09)
Cases	306/486	166/418		141/369	94/312		707/1585
Age groups (y)							
≤24	1.00	1.08 (0.94–1.24)		1.09 (0.93–1.24)	1.12 (0.98–1.11)	.42	1.00 (0.98–1.10)
25–54	1.00	1.07 (0.92–1.21)		1.05 (0.90–1.21)	1.11 (0.95–1.28)	.65	1.00 (0.99–1.02)
55–64	1.00	0.68 (0.45–1.04)		1.09 (0.77–1.56)	1.20 (0.83–1.74)	.04	1.09 (1.02–1.17)
≥65	1.00	0.75 (0.49–1.16)		0.88 (0.56–1.37)	1.47 (0.97–2.16)	.03	1.08 (1.02–1.14)
Elevated cholesterol							
Cases	148/973	178/974		259/974	195/974		780/3895
Model 1	1.00	1.02 (0.85–1.23)		1.08 (0.94–1.13)	1.14 (0.99–1.31)	.08	1.01 (0.98–1.05)
Model 2	1.00	1.02 (0.85–1.23)		1.09 (0.95–1.14)	1.13 (0.99–1.32)	.08	1.02 (0.99–1.05)
Subgroup analysis							
Normoglycemia (n = 1555)	1.00	1.07 (0.96–1.25)		0.93 (0.82–1.10)	1.02 (0.90–1.17)	.32	1.00 (0.99–1.00)
Cases	170/502	114/324		176/361	152/368		612/1555
Prediabetes (n = 1160)	1.00	1.05 (0.92–1.23)		0.93 (0.83–1.08)	1.02 (0.89–1.16)	.33	1.00 (0.99–1.00)
Cases	159/232	107/341		119/287	123/300		508/1160
Diabetes (n = 1180)	1.00	0.99 (0.84–1.13)		1.04 (0.89–1.22)	1.01 (0.87–1.16)	.77	1.00 (0.99–1.02)
Cases	61/307	102/205		133/325	145/343		441/1180
Normal weight (n = 2825)	1.00	0.88 (0.99–1.04)		1.07 (1.00–1.33)	1.19 (1.09–1.41)	.05	1.08 (1.00–1.16)
Cases	298/965	289/520		291/632	262/708		1140/2825
Overweight (n = 426)	1.00	1.12 (0.98–1.28)		1.12 (1.03–1.36)	1.17 (1.07–1.39)	.05	1.08 (1.01–1.18)
Cases	54/88	43/120		52/108	60/110		209/426
Obese (n = 644)	1.00	1.11 (1.00–1.27)		1.12 (1.06–1.25)	1.14 (1.07–1.35)	.05	1.08 (1.01–1.14)
Cases	23/202	28/139		24/161	36/142		111/644
Female (n = 2310)	1.00	1.09 (1.00–1.19)		1.25 (1.06–1.46)	1.22 (1.02–1.35)	.04	1.11 (1.06–1.18)
Cases	263/741	189/580		209/512	220/477		881/2310
Male (n = 1585)	1.00	0.81 (0.65–0.99)		1.09 (0.99–1.22)	1.13 (0.91–1.35)	.05	1.04 (1.00–1.10)
Cases	97/405	169/380		167/399	182/401		615/1585
Age groups (yr)							
≤24	1.00	0.97 (0.88–1.14)		1.002 (0.83–1.24)	1.12 (0.98–1.11)	.38	1.00 (0.97–1.10)
25–54	1.00	1.06 (0.87–1.26)		1.03 (0.94–1.13)	1.13 (0.99–1.29)	.06	0.98 (0.98–1.00)
55–64	1.00	0.99 (0.89–1.13)		1.08 (0.94–1.26)	1.15 (1.00–1.34)	.09	0.99 (0.98–1.01)
≥65	1.00	1.11 (1.00–1.21)		1.31 (1.10–1.51)	1.19 (1.02–1.35)	.04	1.10 (1.03–1.14)

CI = confidence interval, LDL = low-density lipoprotein, OR = odds ratio, SD = standard deviation, SII = systemic immune-inflammation index.

* Data are presented as OR (95% CI).

† Model 1 is adjusted for age and sex. Model 2 is model 1 adjusted for white blood cell count, platelet count, and body mass index.

There was also a significant trend between higher SII and the odds of hypertension (*P* for trend = .04) (Table 2). Across quartiles of SII, there was a significant trend between higher SII and the odds of hypertension in people with diabetes and those aged ≥65 years (*P* for trend = .002, .05, respectively) (Table 2).

The odds of elevated LDL increased with higher SII (OR: 1.07, 95% CI: 1.02–1.14) (model 2; Table 2). This relationship prevailed for subgroups when data were analyzed separately with higher odds of elevated LDL with increasing SII for all groups except for individuals with prediabetes, diabetes, and younger adults aged ≤24 and 25 to 54 years (Table 2).

Across quartiles of SII, there was a significant trend between higher SII and the odds of hypertension (*P* for trend = .0001) (Table 2). Also, there was a significant trend between elevated LDL and SII for several groups including those with normal weight (*P* for trend = .04), overweight (*P* for trend = .05), obesity (*P* for trend = .03), females (*P* for trend = .02), males (*P* for trend = .05), and those aged 55 to 64 and ≥65 years (*P* for trend < .05) (Table 2).

There was no relationship between the odds of elevated cholesterol and higher SII (OR: 1.02, 95% CI: 0.99–1.05) (model 2; Table 2) and across quartiles of SII. However, when data were analyzed separately, there was a significant relationship for some subgroups with higher odds of elevated cholesterol observed with increasing SII for certain groups (overweight, obese, females, males, and those aged ≥65 years) (Table 2).

The tests for trends were significant for some groups including those with normal weight, overweight, obesity, females, males, and those aged ≥65 years (*P* for trend <.05) (Table 2).

## 4. Discussion

To our knowledge, this study represents the first to retrospectively examine the relationship between SII and cardiovascular risk factors (i.e., high blood pressure and dyslipidemia) in a cohort of adults in Saudi Arabia. The main findings suggest a significant relationship between SII and several aspects of dyslipidemia (e.g., cholesterol, LDL-cholesterol) and hypertension. Additional associations were subgroup-specific.

The link between SII and markers of cardiovascular disease is biologically plausible. Cardiovascular disease progression, including developing dyslipidemia and hypertension, includes inflammatory processes and immune responses as vital components.^[36,37]^ Specifically, the oxidative stress caused by inflammation can lead to endothelial dysfunction and microvascular remodeling, which in turn can increase blood pressure,^[36]^ and proinflammatory cytokines encourage the synthesis of triglycerides, stimulating the production of VLDL and can also result in decreased HDL-cholesterol levels.^[37]^

The present study found a relationship between SII and hypertension aligning with prior research.^[28–30]^ Three NHANES studies confirmed a positive relationship between SII and blood pressure.^[28–30]^ In an NHANES examination of metabolic syndrome and SII, elevated blood pressure was significantly and positively linked with SII.^[16]^ The threshold effect of SII on hypertension was determined to be 501.2 from one of the NHANES studies, with individuals over that threshold having higher odds of hypertension.^[28]^ Jin et al^[29]^ found significant differences in diastolic blood pressure between quartiles of logSII but no significant differences in systolic blood pressure. Xu et al^[30]^ used the primary outcome of hypertension but also assessed systolic and diastolic blood pressure levels grouped by antihypertensive medications, for which they found inverse correlations with SII among those being treated.

We found that some aspects of dyslipidemia were associated with SII, which aligns with the results from the prior studies that have examined the relationship between dyslipidemia and SII.^[15,16]^ Mahemuti et al^[15]^ found dyslipidemia and SII were significantly correlated, however, they defined dyslipidemia as a single binary outcome based on lipid profiles or the use of cholesterol-lowering medications rather than the individual aspects of dyslipidemia examined in this study. The covariates of sex, age, smoking, body mass index, and others were not significantly correlated in the relationship, nor did they result in differential outcomes by subgroups in that study.^[15]^ Zhao et al^[16]^ incorporated aspects of dyslipidemia into definitions of metabolic syndrome and found elevated triglycerides and low levels of HDL-cholesterol were more common in higher quartiles of SII. When models were adjusted for covariates, the relationship with HDL-cholesterol, but not with triglycerides, persisted.^[16]^ This varies from the present results, which showed an association between several markers of dyslipidemia in the total population (i.e., total cholesterol, LDL-cholesterol). Differences in study design or population may contribute to the differential findings between the present and previous studies. The previous work has been mainly cross-sectional, pulling from the US NHANES datasets.

The relationships identified in this study varied by age subgroups, particularly regarding SII and hypertension, LDL-cholesterol, and total cholesterol among older adults. There have also been differences in SII in terms of age noted in prior studies, with older ages seen with higher SII.^[16,38]^ Our subgroup analyses also revealed BMI as an important factor in the relationships between blood pressure, hyperlipidemia, and SII, as there were differential outcomes when analyses were stratified by BMI categories. BMI and age both play a key role in the development of systemic inflammation and cardiovascular risk factors.^[39–41]^ Age-associated structural and functional cardiovascular changes can contribute to the increased risk of cardiovascular disease associated with aging.^[41]^ More specifically, results from the Framingham study showed that HDL-cholesterol levels declined with age while levels of total cholesterol along with BMI increased with age until a decline was seen among older adults (≥65 years); these are changes that correspond well with the present findings.^[42]^

In terms of strengths, as a large retrospective cohort study, the sample was big enough to conduct analyses overall and by subgroups. There was also information on medicinal therapies so we could account for them in the analyses. We were also able to avoid self-report bias as diagnoses were based on physician diagnoses.^[43]^ The original intention of this cohort was to investigate the relationship between diabetes markers and other inflammatory markers. As such, the cohort was not specifically designed to answer questions related to SII and cardiovascular disease biomarkers. Due to this, some lifestyle variables related to blood pressure, hyperlipidemia, and SII were unavailable to incorporate into the analyses (e.g., diet and physical activity level). Lastly, this cohort focused on adults in Saudi Arabia, so the findings may not be generalizable to other groups.

In conclusion, our results suggest that SII may provide value in terms of predicting or identifying individuals with cardiovascular disease risk markers (e.g., hypertension, hyperlipidemia). Mitigating the impact of cardiovascular disease in Saudi Arabia is essential from a health and economic standpoint.^[2]^ Future research can confirm these relationships and the predictive utility of SII in prospective cohort studies.

## Author contributions

**Data curation:** Ghadeer S. Aljuraiban, Fahad J. Alharbi, Ali O. Aljohi, Bader Saad Alotaibi.

**Formal analysis:** Ghadeer S. Aljuraiban, Waad Alfawaz.

**Methodology:** Ghadeer S. Aljuraiban, Abdulaziz S. Alsahli, Waad Alfawaz.

**Resources:** Ghadeer S. Aljuraiban, Manal Abudawood.

**Writing—original draft:** Ghadeer S. Aljuraiban.

**Conceptualization:** Abdullah Z. Almeshari, Abdulaziz S. Alsahli, Bader Saad Alotaibi, Manal Abudawood, Mahmoud Abulmeaty.

**Project administration:** Abdullah Z. Almeshari.

**Software:** Manal Abudawood.

**Writing—review & editing:** Manal Abudawood, Mahmoud Abulmeaty.

**Supervision:** Mahmoud Abulmeaty.

## Supplementary Material


