# Supplementary material for: Systemic immune-inflammation index and its relation to blood pressure and dyslipidemia in adults: A retrospective study
Source: Medicine (Baltimore). 2024 Jul 12;103(28):e38810. doi: 10.1097/MD.0000000000038810 (PMC11245260; doi:10.1097/MD.0000000000038810)
Supplement: Supplementary file 1 [file medi-103-e38810-s001.docx]

| **Supplementary Table 1. General characteristics of participants, n= 3,895^a^** | | |
| --- | --- | --- |
| Age (y) | 48.6 | (18.6) |
| Age groups (y) |  |  |
| ≤ 24 | 11.4 |  |
| 25-54 | 48.4 |  |
| 55-64 | 20.8 |  |
| ≥65 | 19.4 |  |
| Male | 40.7 |  |
| BMI (kg/m^2^) | 29.6 | (18.0) |
| Normal weight | 76.0 |  |
| Overweight | 10.0 |  |
| Obese | 14.0 |  |
| SBP (mmHg) | 126.3 | (19.5) |
| DBP (mmHg) | 74.4 | (11.4) |
| Hypertension | 14.0 |  |
| Cholesterol (mmol/L) | 4.6 | (1.1) |
| Triglycerides (mmol/L) | 18.4 | (18.0) |
| High-density lipoprotein (mmol/L) | 1.3 | (0.3) |
| Low-density lipoprotein (mmol/L) | 2.7 | (0.9) |
| Neutrophil 10^2⁄L | 3.7 | (1.8) |
| Platelet 10^2⁄L | 298.9 | (84.1) |
| Lymphocyte 10^2⁄L | 2.5 | (0.9) |
| White Blood Cells 109/L | 7.0 | (2.4) |
| SII | 500.5 | (217.6) |
| FBS (mg/dL) | 52.4 | (34.4) |
| Insulin (pmol/L) | 14.8 | (11.6) |
| HBA1C % | 6.7 | (1.8) |
| Non-diabetic | 32.9 |  |
| Prediabetic | 42.1 |  |
| Diabetic | 25.0 |  |
| ^a^ Data are presented as mean (SD) or %  BMI: Body mass index; DBP: Diastolic Blood Pressure; FBS: Fasting blood sugar; HBA1C: Hemoglobin A1c; SBP: Systolic Blood Pressure; SII: Systemic immune-inflammation index | | |
